# Procedural time reduction associated with active esophageal cooling during pulmonary vein isolation

**DOI:** 10.1007/s10840-022-01204-1

**Published:** 2022-04-13

**Authors:** Christopher Joseph, Jacob Sherman, Alex Ro, Westby G. Fisher, Jose Nazari, Mark Metzl

**Affiliations:** 1 University of Texas Southwestern Medical Center, Dallas, TX 75390 USA; 2 Washington University, 1 Brookings Dr, St. Louis, MO 63130 USA; 3 NorthShore University Health System, Evanston, IL USA

**Keywords:** Procedure duration, Catheter ablation, Esophageal cooling, Atrioesophageal fistula, Esophageal injury

## Abstract

**Background:**

Active esophageal cooling is increasingly utilized as an alternative to luminal esophageal temperature (LET) monitoring for protection against thermal injury during pulmonary vein isolation (PVI) when treating atrial fibrillation (AF). Published data demonstrate the efficacy of active cooling in reducing thermal injury, but impacts on procedural efficiency are not as well characterized. LET monitoring compels pauses in ablation due to heat stacking and temperature overheating alarms that in turn delay progress of the PVI procedure, whereas active esophageal cooling allows avoidance of this phenomenon. Our objective was to measure the change in PVI procedure duration after implementation of active esophageal cooling as a protective measure against esophageal injury.

**Methods:**

We performed a retrospective review under IRB approval of patients with AF undergoing PVI between January 2018 and February 2020. For each patient, we recorded age, gender, and total procedure time. We then compared procedure times before and after the implementation of active esophageal cooling as a replacement for LET monitoring.

**Results:**

A total of 373 patients received PVI over the study period. LET monitoring using a multi-sensor probe was performed in 198 patients, and active esophageal cooling using a dedicated device was performed in 175 patients. Patient characteristics did not significantly differ between groups (mean age of 67 years, and gender 37.4% female). Mean procedure time was 146 ± 51 min in the LET-monitored patients, and 110 ± 39 min in the actively cooled patients, representing a reduction of 36 min, or 24.7% of total procedure time (*p* < .001). Median procedure time was 141 [IQR 104 to 174] min in the LET-monitored patients and 100 [IQR 84 to 122] min in the actively cooled patients, for a reduction of 41 min, or 29.1% of total procedure time (*p* < .001).

**Conclusions:**

Implementation of active esophageal cooling for protection against esophageal injury during PVI was associated with a significantly large reduction in procedure duration.

## Introduction

As the utilization of catheter ablation for the treatment of atrial fibrillation increases, a focus on thermal injury has increased, given the risks associated with radiofrequency ablation [1]. Until the recent introduction of active cooling, luminal esophageal temperature (LET) monitoring had been the standard of care [2]. However, LET often notifies the electrophysiologist after the esophageal temperature has reached dangerous levels—after injury has occurred [3, 4]. Consequently, temperature alarms in ablations that utilize LET monitoring can result in frequent pauses to wait for luminal temperature to return to safe levels. These pauses lead to increased procedure times and suboptimal ablations given an increase in the continuity index of each ablation [5, 6]. Reducing procedure time can also reduce complications such as postoperative cognitive dysfunction [7].

Active cooling appears to reduce the risk of severe esophageal injury [8–10]. To date, despite thousands of uses of active cooling (currently over 8500), no atrioesophageal fistula has yet been reported with active esophageal cooling [11]. Hypothesized mechanisms for this effect extend beyond dissipation of heat, and include the prevention of esophageal wall temperatures from reaching lethal isotherm temperatures and the mitigation of the inflammatory cascade contributing to burn progression [12–15]. Because active cooling eliminates overheating and avoids temperature alarms, active cooling also allows electrophysiologists to operate without pauses and unnecessary time delays [14, 16, 17]. In order to quantify this effect, we measured procedure lengths at our single center two-hospital system and compared procedure times before and after the introduction of active esophageal cooling as a means for reducing esophageal thermal injury.

## Methods

### Study design and population

This study was a retrospective review under IRB approval of all patients with atrial fibrillation who were treated with left atrial RF ablation by two physicians during the period January 2018 to February 2020. No patients fitting these criteria were excluded. Patients having first-time or re-do ablations were included, and AF types included paroxysmal, persistent, and long-standing persistent. During the time period included in this retrospective study, operators used LET monitoring before February 2019, and then used active esophageal cooling after February 2019 as methods of esophageal protection. To assess whether active esophageal cooling was associated with reduced procedure duration, we compared the procedure times during the LET monitoring period (January 2018–January 2019) with the procedure times during the active esophageal cooling period (February 2019–February 2020) included in this study.

### Data collection and definition

For each patient, we obtained and recorded the total procedure time as recorded in the electronic medical record or existing practice records maintained by the physician practice group. Total procedure time was defined as the time from the “time out” procedure marking the beginning of the case to the time of sheath removal.

### Ablation procedure

Two electrophysiologist physicians performed primarily wide area circumferential pulmonary vein isolation with additional posterior wall isolation as needed. The posterior wall was isolated using a combination of roof and floor linear lesions, along with additional lesions to further segment the posterior wall to achieve entrance and exit block (using output of 20 mA and 5 ms pulses). Patients received general anesthesia for the ablation procedure. Anticoagulation was administered prior to ablation with a heparinized target activated clotting time (ACT) of greater than 300 s. In the right femoral vein, a transseptal catheter, decapolar coronary sinus catheter (Webster CS Bidirectional, Biosense Webster, Inc., Diamond Bar, CA, USA), and intracardiac echocardiographic (ICE) catheter (Soundstar, Biosense Webster, Inc., Diamond Bar, CA, USA) were placed. A very low to no fluoroscopy protocol was followed for all procedures. A 3D geometry was created using the Carto system (Biosense Webster, Inc., Diamond Bar, CA, USA). A single transseptal puncture was performed under ICE and electroanatomic mapping guidance. Electroanatomic mapping, vein voltage, and pace mapping were performed using a multipolar mapping catheter (PentaRay, Biosense Webster, Inc., Diamond Bar, CA, USA).

For ablation, an externally irrigated ablation catheter (ST/SF™, Biosense Webster, Inc., Diamond Bar, CA, USA) was used in all cases. The pulmonary veins were isolated by delivery of RF applications circumferentially to the antral regions to produce a minimum of entrance and exit block for at least 20 min. A Smartablate™ generator (Biosense Webster, Inc.™, Diamond Bar, CA, USA) was used to deliver RF energy, with a setpoint of 50 W on all patients and all areas of the left atrium. The Visitag Surpoint™ module (ablation index) was utilized during ablations, with a target of 400 units on the posterior wall, and 550 units on the anterior wall, lateral wall, and septum and an intertag distance of less than 6 mm. Catheter tip temperature, power, and impedance were recorded for each RF energy application. The operators’ approach to left atrial ablation is dictated primarily by the type of AF the patient presents with, such that essentially, all patients with paroxysmal AF receive only pulmonary vein isolation without additional posterior wall ablation, whereas patients with persistent AF almost all receive additional posterior wall isolation. The operators’ posterior wall method is to place lines with homogenization until exit block throughout at highest output.

### Esophageal protection

During procedures performed between January 2018 and January 2019, operators utilized LET monitoring using a multi-sensor probe (Circa S-Cath™, Circa Scientific, Inc., Englewood, CO, USA). RF ablation was stopped and the site of ablation moved to a different distant area of the veins for any alarm over 0.2 °C/s, or a temperature exceeding 38.5 °C. During procedures performed between February 2019 and February 2020, in which operators used active esophageal cooling, no temperature probe was utilized in PVI cases, and ablation proceeded in a point-to-point fashion uninterrupted by pauses or alarms. Active esophageal cooling was performed using the ensoETM device (Attune Medical, Chicago, IL, USA), a single-use silicone tube with a closed-loop system that is inserted into the esophagus. The device is connected to a temperature controlled heat-exchanger and circulates 4 °C distilled water at 2.4 L/min. Except for the change to the esophageal cooling protocol in the treatment group, the ablation procedure for patients in both groups was the same. Setting up the active cooling system is done in parallel with patient preparation, including anesthesia induction, and therefore does not have a significant (or any) contribution to overall procedure time.

### Statistical analysis

Data were analyzed with IBM SPSS Statistics version 26 (IBM, Armonk, NY), with descriptive statistics (mean, median, and standard deviations), and comparison between groups with the Mann–Whitney *U* test is reported.

## Results

### Baseline characteristics

A total of 373 patients received PVI over the study period. LET monitoring using the multi-sensor probe was performed in 198 patients, and active esophageal cooling was performed in 175 patients. Patient characteristics did not significantly differ between groups (Table 1). The mean age of LET-monitored patients was 67 ± 11 years while the mean age of actively cooled patients was 69 ± 10 years. Patient gender was 36.9% female in the LET-monitored group, and 39.2% female in the actively cooled group. Type of AF did not differ significantly between groups, as shown in Table 1. Anti-arrhythmic drug use was ascertained for a subset of patients in which the data were available, showing in LET-monitored and actively cooled cohorts the use of amiodarone in 10% and 17%, dofetilide in 2.9% and 2.9%, dronedarone in 1.4% and 1.4%, flecainide in 5.7% and 14.3%, and sotalol in 1.4% and 0%, respectively.

**Table 1:** Patient baseline characteristics, including age, gender, and AF type. Those listed under the category “Other” include long-standing persistent AF, atypical flutter, and atrial tachycardia. *LET*, luminal esophageal temperature

		Esophageal protection		*p*-value
		LET monitored	Actively cooled	
Patient age (years), mean (SD)		67.2 ± 11.2	66.8 ± 10	0.62
Gender	Male	63%	61%	0.84
	Female	38%	39%	0.84
AF type	Paroxysmal AF (*n*, %)	85, 43%	99, 57%	0.3
	Persistent AF (*n*, %)	87, 44%	55, 31%	0.3
	Long-standing persistent (*n*, %)	13, 7%	15, 9%	0.3
	LA macro-reentry (*n*, %)	2, 1%	0, 0%	0.3
	Atrial tachycardia (*n*, %)	6, 3%	3, 2%	0.3
	Unknown (*n*, %)	5, 3%	3, 2%	0.3

### Procedural characteristics and association between active esophageal cooling and procedure time

**Fig. 1:**
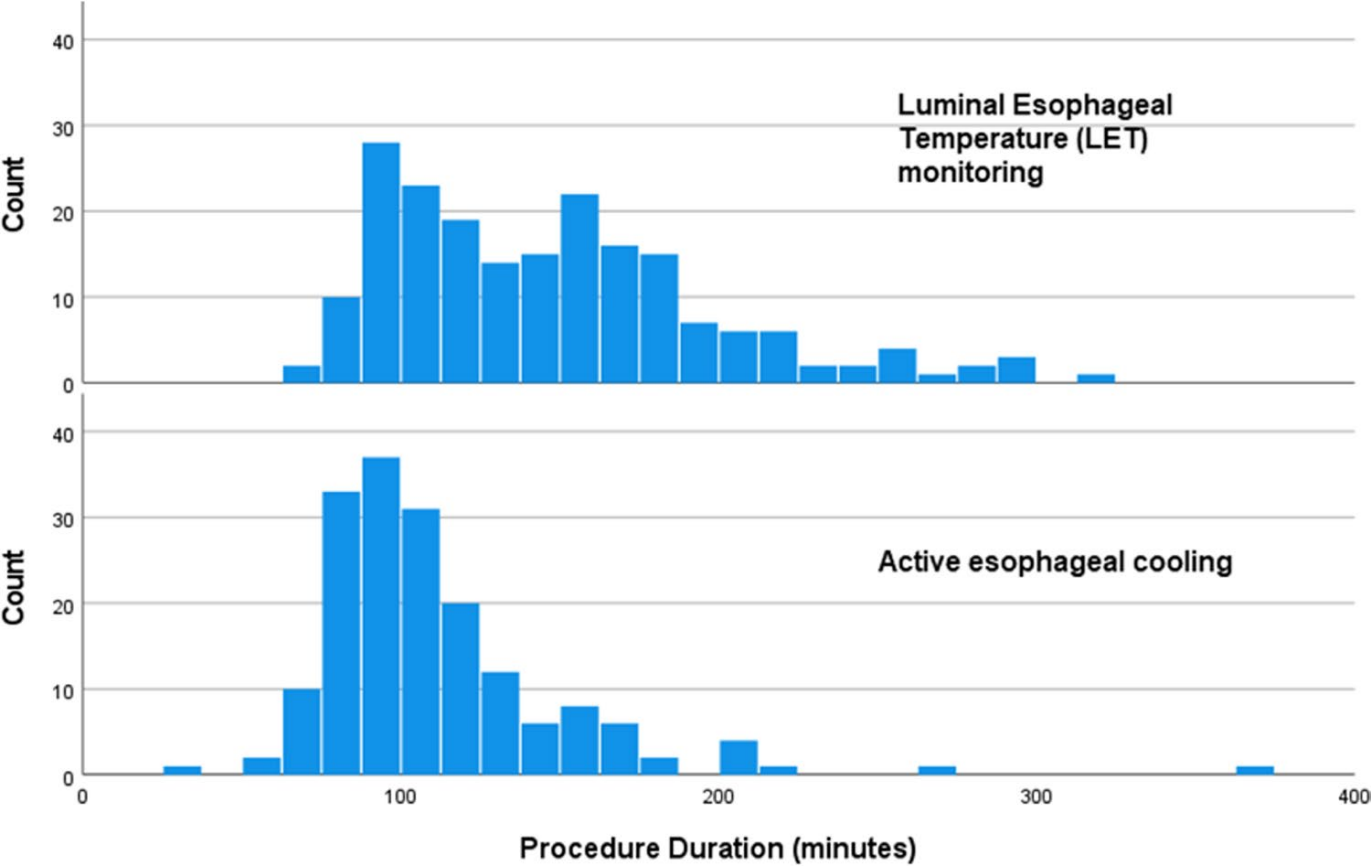
Histogram of procedure times for each of the two cohorts totaling 373 patients (198 received LET monitoring using a multi-sensor probe, and 175 received active esophageal cooling). The top histogram depicts procedure times for LET-monitored patients, and bottom histogram depicts procedure times for actively cooled patients

Patients with paroxysmal AF received only pulmonary vein isolation without additional posterior wall ablation, whereas patients with persistent AF almost all receive additional posterior wall isolation. Patients with left-sided AT receive PVI plus posterior box. Mean procedure time was 146 ± 51 min in the LET-monitored patients, and 110 ± 39 min in the actively cooled patients, representing a reduction of 36 min, or 24.7% (*p* < 0.001). Median procedure time was 141 [IQR 104 to 174] min in the LET-monitored patients and 100 [IQR 84 to 122] min in the actively cooled patients, for a reduction of 41 min, or 29.1% (*p* < 0.001). Figure 1 shows a histogram of procedure times for each of the two cohorts (LET monitored and actively cooled).

Comparison of procedure duration and fluoroscopy time utilization between cohorts is shown in Table 2, where it can be seen that in addition to a shorter procedure duration associated with active cooling, a reduction in fluoroscopy time was associated with the use of active esophageal cooling.

**Table 2:** Procedural characteristics, including total procedure duration (in minutes) and fluoroscopy time (in minutes). *LET*, luminal esophageal temperature

		Esophageal protection		*p*-value
		LET monitored	Actively cooled	
Procedure duration (min), mean (SD)	Overall cohort (*n* = 373)	146 ± 51	110 ± 39	< 0.001
	Paroxysmal AF (*n* = 188)	143 ± 98	102 ± 34	< 0.001
	Persistent AF (*n* = 147)	153 ± 51	119 ± 47	< 0.001
Fluoroscopy time (min), mean (SD)	Overall cohort (*n* = 373)	2.7 ± 6.6	1.6 ± 3.2	0.001
	Paroxysmal AF (*n* = 188)	3.6 ± 5.5	1.9 ± 3.7	< 0.001
	Persistent AF (*n* = 147)	2.4 ± 8.2	0.7 ± 1.8	0.027

For a subset of 172 patients for which data were available, radiofrequency ablation duration was 24.0 min for LET-monitored patients, and 20.6 min for actively cooled patients, while the number of RF lesions was 61 for LET-monitored patients, and 46 for actively cooled patients. Of the LET-monitored procedures, 15% were re-do ablations, while 8% of the actively cooled procedures were re-do ablations (Table 3).

**Table 3:** Analysis of additional procedure characteristics and anti-arrhythmic drug use for the subset of patients in which data were available

	Esophageal protection	
	LET monitored (*n* = 75)	Cooled (*n* = 97)
Procedure duration (min), mean (SD)	159 ± 52	113 ± 46
Fluoroscopy duration (min), mean (SD)	0.7 ± 1.6	0.6 ± 1.8
RF duration (min), mean (SD)	24 ± 9.9	21 ± 11
# of RF lesions, mean (SD)	63 ± 34	47 ± 28
Prescribed anti-arrhythmic drugs	16 (21.4%)	34 (35.6%)

Analysis of provider-specific procedural times showed similar reductions in procedure times with active cooling. One operator had a mean procedure duration of 142 ± 52 min with LET monitoring, and 112 ± 32 min with active esophageal cooling. The other operator had a mean procedure duration of 151 ± 50 min with LET monitoring, and 109 ± 41 min with active esophageal cooling.

## Discussion

In this largest study to date on the impact of active esophageal cooling on procedure duration, a significant reduction in time was evident after the implementation of active cooling as an esophageal protective strategy. A mean procedure time reduction of 36 min was seen, representing a 24.7% reduction from baseline, and a median procedure time reduction of 41 min was seen, representing a 29.1% reduction from baseline. In both measures of procedure duration, a procedure time of under 2 h was attained after converting to active esophageal cooling from LET monitoring during PVI cases (mean of 110 min, and median of 100 min).

Procedure times for PVI with RF ablation will naturally vary by operator and site, but recent studies have reported times of 132 to 188 min for RF ablations utilizing a variety of esophageal protection techniques (single-sensor LET monitoring, multi-sensor LET monitoring, or power reduction on the posterior wall) [18–21]. As such, the reductions seen in this study are notable. The likely mechanism for this reduction stems from the elimination of pauses in ablation that are compelled by LET sensor alarms and concerns over heat stacking. Alarm sensitivity can be adjusted on some systems, raising or lowering the sensitivity of measurements. The settings utilized during LET monitoring prior to the adoption of active esophageal cooling were such that alarms were triggered, and RF ablation was stopped (with the site of ablation moved to a different distant area) at a temperature rise of 0.2 °C/s or greater, or a temperature exceeding 38.5 °C. Although these are fairly typical settings, higher thresholds have been used by other groups, which in turn may influence results (reducing the number of pauses required, at the cost of increased risk of esophageal injury) [19].

Despite shortcomings in protective effects, LET continues to be the standard of care in many electrophysiology labs across the world. Recent studies, including randomized controlled and prospective interventional studies, have raised concerns that LET monitoring does not reduce, and may actually increase, esophageal lesion formation [18, 19, 22]. Proposed mechanisms for this finding involve physical limitations in adequately positioning temperature sensors to detect thermal insults, and inherent limitations in detecting temperature rise before damage has occurred [3, 4]. In contrast, three randomized controlled studies have demonstrated benefits with active esophageal cooling, with the largest study of 120 patients showing reductions of all lesion formation of 83%, and reductions of severe lesion formation of 100% on per-protocol analysis [8, 9, 23]. Shortening of the procedure time may provide further incentive to transition from a passive monitoring strategy to an active cooling strategy for esophageal protection. In addition to reducing lab costs and improving staff satisfaction, shorter procedure times may also reduce postoperative complications such as postoperative cognitive dysfunction [7]. Beyond the acute benefits, long-term efficacy improvement has also been suggested with active cooling, with trends towards greater freedom from AF at 12 months after active cooling than with LET monitoring in randomized controlled data, and significant improvements shown in various single-site reviews have recently been presented [17, 24, 25]. This is hypothesized to be due to the lower continuity index that can be obtained with active cooling, allowing consistent, contiguous placement of lesions without the pauses and repositioning required with LET monitoring [6].

The largest randomized controlled trial of active esophageal cooling to date is the IMPACT study, which found an 83% reduction in all grades of esophageal injury on endoscopy with cooling, and a 100% reduction in severe grade lesions on a per-protocol analysis [9]. Although a procedure duration reduction of 8 min was found with active esophageal cooling in the IMPACT study, the baseline procedure time was high in the academic medical center in which this study was performed. Because of the additional time incorporated in procedure performance in an academic institution, where procedures are generally performed by fellows in training, a lower bound on procedure time is likely fixed by the necessary prioritization of teaching operators-in-training.

A recent study found a 35% reduction in fluoroscopy usage after the implementation of active cooling (compared to patients treated with single-sensor LET monitoring) [16]. Zagrodzky et al. did not report shorter procedure times, but their procedure durations were sufficiently short that further significant shortening may have been unachievable. In the current analysis, a significant reduction in fluoroscopy usage was also seen, in line with the earlier findings of Zagrodzky et al. [16]. Notably, these earlier findings were identified in a comparison between single-sensor LET monitoring and active cooling, whereas the current data compare multi-sensor LET monitoring to active cooling.

We have not provided long-term follow-up data on patients in this analysis but have completed a separate analysis on this outcome for a separate publication with the focus on the outcome of 1-year freedom from arrhythmia specifically. The data show improved freedom from atrial arrhythmias at 1 year in patients treated with active esophageal cooling, in agreement with data from two other sites utilizing active esophageal cooling that have recently been presented, showing improved freedom from atrial arrhythmias at 1 year [24, 25]. This is presumed to be due to the improved lesion continuity attainable with active cooling, which has been shown to improve long-term success in maintaining isolation and reducing AF recurrence [5, 6].

The primary reason for a reduction in procedure time is presumed to be due to the elimination of (a) the need to pause RF ablation due to high-temperature alarms, and (b) the need to stop to reposition a temperature sensor opposite the RF catheter tip to ensure adequate identification of temperature elevations. Since this is a retrospective analysis, the count of high-temperature alarms, the number of catheter movements, and the amount of temperature sensor repositioning are not available, as these data are not typically recorded. Nevertheless, in the operators’ experience, a typical number of alarms would be 3–10 for paroxysmal AF ablations, and 6–20 for persistent AF ablations. All of these high-temperature alarms induce the operator to cease RF power at that site. At least half of these will result in skipping to another location in the atrium, and in the remainder, the operator will typically wait for equilibration and a return to baseline temperature. The wait time for temperature equilibration between consecutive posterior wall lesions after a high esophageal temperature alarm ranges on the order of minutes. When active esophageal cooling is utilized, none of these interruptions occurs, and additionally, no stopping is required to reposition the active cooling device.

### Limitations

While this study demonstrates an association between active cooling and reduced overall procedure time, we are unable to definitively conclude that the reduction of procedure time is due to the use of active cooling. No significant procedural changes were made during the period of observation other than the switch to active esophageal cooling from LET monitoring (patients received either LET monitoring, before February 2019, or active esophageal cooling without any LET monitoring after February 2019), which lends support to a causative effect from active esophageal cooling. Given the experience level of the operators (more than 10 years out from completion of fellowship), we believe most of the time efficiencies in routine portions of the procedure have been well optimized already. We were not able to quantify the number of pauses that occurred during ablations utilizing LET monitoring, since these are typically not captured in the medical record. Additionally, given the use of multi-sensor temperature probes in this study, the aforementioned procedure time benefit may not be applicable to labs that utilize single-sensor probes. This study did not perform evaluation of the esophagus following ablation; however, abundant data exist demonstrating the safety of this approach and therefore, the risk of additional endoscopy of these patients would outweigh benefits. To date, with thousands of uses of active cooling during ablation with no atrioesophageal fistulas reported, the safety appears to be substantially greater than any alternative modalities [2, 9, 11, 16]. We did not assess long-term clinical outcomes of patients in this study; however, randomized data to date suggest no degradation in procedural efficacy, and in fact suggest trends towards improved efficacy with the use of active cooling. Recently presented data on 168 patients found that freedom from AF at 1 year was 71.8% for LET-monitored patients and 93.0% for actively cooled patients, representing an absolute increase in freedom from AF of 14% with active esophageal cooling (*p* = 0.045) [24]. Recently presented data on 544 patients from a different single center found 1-year KM estimates for freedom from arrhythmia were 41.5% for LET-monitored patients, and 64.3% for patients receiving active esophageal cooling (*p* < 0.001) [25]. A large multicenter analysis of over 500 patients in preparation by the authors has identified a statistically significant 14% improvement in freedom from atrial fibrillation at 1-year follow-up [26]. This effect is believed to be due to enhanced facilitation of point-to-point lesion placement and the known efficacy improvement that results from a lower continuity index [5, 6]. In order to collect a large enough data set for analysis, we identified two electrophysiologists who were the earliest adopters of active esophageal cooling. We then reviewed data from procedures performed by these operators to have comparable LET monitoring and active cooling cohorts. Although the two remaining physicians in the group who have more recently adopted active cooling also report shortened procedure times, quantification of these differences has not yet been completed. Although we do not have some additional patient factors available for all patients, such as left atrial (LA) size or CHA2DS2-VASc scores, a review of these data from available records does not suggest any changes in the preceding years, with median LA size of 42 mm [IQR 38 to 47] and median CHA2DS2-VASc score of 2 [IQR 1 to 4]. Because of the retrospective nature of this study, the time to reach specific index targets is not available; however, we have not seen an increase in time to form lesions, or reach the target AI when using active cooling. This observation is further supported in the fact that rather than increase, total RF time decreased slightly when using active cooling.

## Conclusion

Implementation of active esophageal cooling for protection against esophageal injury during PVI was associated with a significant and large reduction in procedure duration.

## References

[1] Della Rocca DG, Magnocavallo M, Natale VN, Gianni C, Mohanty S, Trivedi C, Lavalle C, Forleo GB, Tarantino N, Romero J (2021). Clinical presentation, diagnosis, and treatment of atrioesophageal fistula resulting from atrial fibrillation ablation. J Cardiovasc Electrophysiol.

[2] Leung LWM, Akhtar Z, Sheppard MN, Louis-Auguste J, Hayat J, Gallagher MM: Preventing esophageal complications from atrial fibrillation ablation: a review. *Heart Rhythm O2* 2021, **2**(6Part A):651–664 10.1016/j.hroo.2021.09.004 PMID: 34988511. 10.1016/j.hroo.2021.09.004 PMC8703125 34988511

[3] Kar R, Post A, John M, Rook A, Razavi M (2021). An initial ex vivo evaluation of temperature profile and thermal injury formation on the epiesophageal surface during radiofrequency ablation. J Cardiovasc Electrophysiol.

[4] Barbhaiya CR, Kogan EV, Jankelson L, Knotts RJ, Spinelli M, Bernstein S, Park D, Aizer A, Chinitz LA, Holmes D (2020). Esophageal temperature dynamics during high-power short-duration posterior wall ablation. Heart Rhythm.

[5] Jankelson L, Dai M, Aizer A, Bernstein S, Park DS, Holmes D, Chinitz LA, Barbhaiya C (2021). Lesion sequence and catheter spatial stability affect lesion quality markers in atrial fibrillation ablation. JACC Clinical electrophysiology.

[6] Kautzner J, Neuzil P, Lambert H, Peichl P, Petru J, Cihak R, Skoda J, Wichterle D, Wissner E, Yulzari A (2015). EFFICAS II: optimization of catheter contact force improves outcome of pulmonary vein isolation for paroxysmal atrial fibrillation. Europace.

[7] Medi C, Evered L, Silbert B, Teh A, Halloran K, Morton J, Kistler P, Kalman J (2013). Subtle post-procedural cognitive dysfunction after atrial fibrillation ablation. J Am Coll Cardiol.

[8] Tschabrunn CM, Attalla S, Salas J, Frankel DS, Hyman MC, Simon E, Sharkoski T, Callans DJ, Supple GE, Nazarian S (2021). Active esophageal cooling for the prevention of thermal injury during atrial fibrillation ablation: a randomized controlled pilot study. J Interv Card Electrophysiol.

[9] Leung LWM, Bajpai A, Zuberi Z, Li A, Norman M, Kaba RA, Akhtar Z, Evranos B, Gonna H, Harding I (2021). Randomized comparison of oesophageal protection with a temperature control device: results of the IMPACT study. Europace.

[10] Leung LWM, Akhtar Z, Sheppard MN, Louis-Auguste J, Hayat J, Gallagher MM: Preventing esophageal complications from atrial fibrillation ablation: a review. *Heart Rhythm O2*10.1016/j.hroo.2021.09.004 PMID:10.1016/j.hroo.2021.09.004 PMC8703125 34988511

[11] Leung L, Bajpai A, Zuberi Z, Li A, Norman M, Kaba R, Sohal M, Akhtar Z, Evranos B, Gonna H *et al*: A registry review of 2532 catheter ablations for atrial fibrillation using active thermal protection. *EP Europace* 2021, **23**(Supplement_3) 10.1093/europace/euab116.250 PMID:

[12] Griffin BR, Frear CC, Babl F, Oakley E, Kimble RM (2020). Cool running water first aid decreases skin grafting requirements in pediatric burns: a cohort study of two thousand four hundred ninety-five children. Ann Emerg Med.

[13] Rizzo JA, Burgess P, Cartie RJ, Prasad BM (2013). Moderate systemic hypothermia decreases burn depth progression. Burns.

[14] Mercado M, Leung L, Gallagher M, Shah S, Kulstad E (2020). Modeling esophageal protection from radiofrequency ablation via a cooling device: an analysis of the effects of ablation power and heart wall dimensions. Biomed Eng Online.

[15] Wright EH, Harris AL, Furniss D (2015). Cooling of burns: mechanisms and models. Burns.

[16] Zagrodzky J, Bailey S, Shah S, Kulstad E: Impact of active esophageal cooling on fluoroscopy usage during left atrial ablation. *J Innov Card Rhythm Manag* 2021, **12**(11):4749–4755 10.19102/icrm.2021.121101 PMID: 34676132. 10.19102/icrm.2021.121101 PMC8519316 34676132

[17] Leung L, El Batran A, Dhillon G, Bajpai A, Zuberi Z, Li A, Norman M, Kaba R, Akhtar Z, Evranos B *et al*: Oesophageal thermal protection during AF ablation: effect on left atrial myocardial ablation lesion formation and patient outcomes. *EP Europace* 2021, **23**(Supplement_3) 10.1093/europace/euab116.253 PMID:

[18] Schoene K, Arya A, Grashoff F, Knopp H, Weber A, Lerche M, König S, Hilbert S, Kircher S, Bertagnolli L (2020). Oesophageal Probe Evaluation in Radiofrequency Ablation of Atrial Fibrillation (OPERA): results from a prospective randomized trial. Europace.

[19] Grosse Meininghaus D, Blembel K, Waniek C, Kruells-Muench J, Ernst H, Kleemann T, Geller JC (2021). Temperature monitoring and temperature-driven irrigated radiofrequency energy titration do not prevent thermally induced esophageal lesions in pulmonary vein isolation: a randomized study controlled by esophagoscopy before and after catheter ablation. Heart Rhythm.

[20] Verma A, Jiang CY, Betts TR, Chen J, Deisenhofer I, Mantovan R, Macle L, Morillo CA, Haverkamp W, Weerasooriya R (2015). Approaches to catheter ablation for persistent atrial fibrillation. N Engl J Med.

[21] Loring Z, Holmes DN, Matsouaka RA, Curtis AB, Day JD, Desai N, Ellenbogen KA, Feld GK, Fonarow GC, Frankel DS *et al*: Procedural patterns and safety of atrial fibrillation ablation. *Circulation: Arrhythmia and Electrophysiology* 2020, **13**(9):e007944 doi:10.1161/CIRCEP.119.007944 PMID:10.1161/CIRCEP.119.007944 PMC7502261 32703018

[22] Chen S, Schmidt B, Seeger A, Bordignon S, Tohoku S, Willems F, Urbanek L, Throm C, Konstantinou A, Plank K (2020). Catheter ablation of atrial fibrillation using ablation index-guided high power (50 W) for pulmonary vein isolation with or without esophageal temperature probe (the AI-HP ESO II). Heart Rhythm.

[23] Clark B, Alvi N, Hanks J, Suprenant B: A pilot study of an esophageal cooling device during radiofrequency ablation for atrial fibrillation. *medRxiv* 2020:2020.2001.2027.20019026 10.1101/2020.01.27.20019026 PMID:

[24] Zagrodzky J, Zagrodzky W, Joseph C, Bailey S, Kulstad E: Proactive esophageal cooling during radiofrequency ablation is associated with improved one-year freedom from atrial fibrillation. In: *AF Symposium 2022.* New York, NY; 2022.

[25] Joseph C, Francisco G, Ruppert A, Willms D, Athill C: Active esophageal cooling is associated with improved freedom from arrhythmias at one-year: a large hospital registry review. In: *AF Symposium 2022.* New York, NY; 2022.

[26] Metzl M, Nazari J, Zagrodzky J, Sherman J, Zagrodzky W, Joseph C, Bailey S, Kulstad E: One-year outcomes after active cooling during left atrial radiofrequency ablation. In: *ACC 2022.* Washington D.C. ; 2022. 10.1007/s10840-023-01474-3 PMC10359433 36670327

